# Screening Traditional Foods for the Prevention of Enterotoxigenic *Escherichia coli* K88ac (F4ac) Attachment to IPEC-J2 Cells

**DOI:** 10.3390/foods13060952

**Published:** 2024-03-21

**Authors:** Yanan Zhu, Changyan Shao, Susana María Martín-Orúe

**Affiliations:** 1 School of Food and Biological Engineering, Shaanxi University of Science and Technology, Xi’an 710021, China; 2 Animal Nutrition and Welfare Service, Animal and Food Science Department, Universitat Autònoma de Barcelona, 08193 Bellaterra, Spain

**Keywords:** highland barley, shiitake, ETEC K88ac, adhesion, intestine

## Abstract

Enterotoxigenic *Escherichia coli* (ETEC) is the major diarrhoea-causing pathogen world-wide. Fimbria–receptor recognition is the primary step when attachment of ETEC to the intestine occurs. This study aims to evaluate the potential of some traditional foods, particularly those rich in β-glucans, as analogues for fimbriae or receptors in reducing ETEC colonisation. The adhesion test (AT) demonstrated that aqueous extracts of highland barley (EHB), black rice (EBR) and little millet (ELT) at concentrations of 2% and 1% could attach to more ETEC K88ac (*p* < 0.001), as well as aqueous extracts of shiitake (EST) (*p* < 0.01). The competition test (CT) revealed that EHB and EST significantly prevented ETEC K88ac from adhering to intestinal epithelial cells (IPEC-J2) at 2% (*p* < 0.01) and 1% (*p* < 0.05). In the Exclusion Test (ET) and the displacement test (DT), the food samples were unable to impair ETEC colonisation in terms of blocking receptors or removing attached pathogens. These results demonstrate how some traditional foods such as highland barley and shiitake contain bioactive compounds that interfere with the attachment of ETEC to the intestinal epithelium, and their potential in the prevention and treatment of ETEC diarrhoea.

## 1. Introduction

Diarrhoea induced by Enterotoxigenic *Escherichia coli* (ETEC) causes more than 300,000 paediatric deaths and millions of clinical cases every year [1,2]. The virulence factors of ETEC, adhesins and enterotoxins, are responsible for clinical diarrhoea [3]. The recognition between ETEC fimbriae and receptors on intestinal cell surfaces is the primary step when colonising hosts, and the pathogen subsequently produces and excretes enterotoxins, leading to water diarrhoea [4,5]. Providing exogenous fimbriae or/and host receptor analogues, especially those from natural ingredients, was considered an ecological and competition-based way to prevent *E. coli*-induced diarrhoea [6].

Traditionally, ingredients or food have been considered to just provide energy, proteins, minerals and vitamins. However, the functional properties of these ingredients also play crucial roles in improving the health of humans and animals. In recent years, different studies have shown the ability of common ingredients to impair the attachment of ETEC K88ac to the intestinal epithelium [6,7]. Specifically, it has been shown that some common cereals, such as wheat, oat, rye and cereal by-products, can decrease the adhesion of pathogens to intestinal cells when using miniaturised in vitro models [8]. Many of these ingredients are rich in β-glucans, suggesting that this fraction may be responsible for the anti-adhesive properties [9].

In the literature, glucans have been described as analogues disrupting the adhesion of *E. coli* fimbriae to the host receptors, which could ultimately help to prevent intestinal bacterial disorders. For examples, Stuyven et al. (2009) found that weaning piglets receiving β-glucans from *Saccharomyces cerevisiae* and *Sclerotium rolfsii* had fewer F4^+^ ETEC infections accompanied by changes in the blood concentration of erythrocytes and leukocytes after an oral challenge with the pathogen [10]. In the research reported by Kšonžeková et al. (2016), β-glucans produced by *Lactobacillus reuteri* strains also reduced the ETEC attachment to intestinal epithelial cells in vitro [11]. Heteropolysaccharides from the *S. cerevisiae* cell wall, which mainly contain glucan and mannan, have also been shown to reduce *E. coli* adhesiveness in vitro and in vivo [12]. These findings have encouraged the search for functional foods, especially those rich in β-glucans, such as highland barley, shiitake and jelly ear, although the physicochemical properties of these compounds may vary between different food sources [13,14,15,16,17]. Additionally, several studies have explored the potential biological activity of these foods, particularly their influence on the composition of the intestinal microbiota, due to their high glucan content [18,19].

Based on previous research, the hypothesis of this study was that traditional foods with high glucan contents may have anti-adhesive abilities against intestinal pathogens. The objective of this study was to determine whether soluble extracts of five traditional foods (highland barley, shiitake, jelly ear, black rice and little millet) were able to inhibit the adherence of ETEC K88ac to IPEC-J2 cells.

## 2. Materials and Methods

### 2.1. Food Ingredients and Analytical Procedures

The ingredients used in this study were *Gramineae* cereals (highland barley, little millet, black rice) and mushrooms (shiitake, jelly ear). The highland barley was purchased from Sichuan province, while the other ingredients were purchased from Chinese supermarkets. As in previous studies, casein glycomacropeptide (CGMP) (Arla Foods, Viby J., Denmark) was used as the positive control [8]. Phosphate-buffered saline (PBS)-soluble extracts (PSEs, *w*/*v*) were prepared according to Zhu et al. (2018) [8]. All ground samples were re-suspended in PBS at a solid-to-liquid ratio of 1:10 (*w*/*v*) following three vortex and sonication steps. After they underwent centrifugation at 460× *g* for 5 min, all supernatants were finally filtered and stored at −20 °C until use. The analysis of the chemical compositions of the tested ingredients was also carried out, following the methods described in previous papers [20,21]. The chemical compositions of ingredients are presented in Table 1.

### 2.2. Escherichia coli Strains

In this research, an ETEC K88ac (F4ac) strain and a non-fimbriated *E. coli* strain (NF-*E. coli*) were used [8,22]. The ETEC K88ac (F4ac) strain was kindly donated by the *E. coli* Reference Laboratory, Veterinary Faculty of Santiago de Compostela (Lugo), with serotype (O149:K91; H10(K-88)/LT-I/STb). The non-fimbriated *E. coli* (F4-, F6-, F18-, LT1-, ST2+, Stx2e-) was generously provided by the Departament de Sanitat i d’Anatomia Animals of the Universitat Autonoma de Barcelona and served as a control strain without F4, F6 or F18 fimbriae. The preparation procedure for *E. coli* was the same method as that reported by González-Ortiz et al. [7].

### 2.3. Cell Culture Growth

The IEPC-J2 cells, which were isolated from the jejunum of neonatal piglet and expressed receptors for ETEC K88ac (F4ac), were kindly donated by Dr Antony Blikslager from North Carolina State University, Raleigh, NC, USA. The details of the cell culture were as described in the previous research [8].

### 2.4. Adhesion Test

The adhesion test was performed according to previous studies by our group [7]. Briefly, 300 µL of PSEs containing different ingredients was added into 96-well high-binding polystyrene microtitration plates followed by incubation at 4 °C overnight. After a sterile PBS wash, the attached PSEs were incubated with PBS containing 1% bovine serum albumin and 0.5% sodium azide at 4 °C for 1 h. After another two sterile PBS washes, 300 µL of each *E. coli* strain suspension (*E. coli*-Susp) was incubated in the wells for 0.5 h at room temperature. The non-attached bacteria were removed using three PBS washes followed by the addition of 300 µL of Luria broth. The sigmoidal growth of *E. coli* was monitored at 650 nm, as in previous studies [23].

### 2.5. Miniaturised Assays with IPEC-J2 Cells

Assays using IPEC-J2 cells were conducted as previously described [7,24]. Firstly, the IPEC-J2 monolayer was incubated with a CO_2_-independent medium for 24 h at 37 °C prior to being used in the miniaturised assays. The *E. coli*-Susp (OD = 1) was prepared at a 1:100 dilution to optimise the ratio of bacteria/cells. All of the following steps were performed gently to avoid disturbing the cell monolayer.

- Competition Test

In the CT, PSEs were first mixed with the same volume of each *E. coli*-Susp. The cell monolayers were then incubated with these mixtures at 200 µL volume at 37 °C for 0.5 h, which would allow the non-blocked *E. coli* to attach to the cells. One PBS wash was gently performed to remove any non-adhered *E. coli*.

b. Exclusion Test

In the ET, PSEs were first mixed with the same volume of PBS followed by incubation with cell monolayers in 200 µL volume at 37 °C for 0.5 h, which allowed PSEs to attach to the cells. Two PBS washes were performed to remove any non-adhered PSEs. Suspensions of *E. coli* strains were also mixed with the same volume of PBS and then 200 µL of these mixtures was incubated with cells at 37 °C for 0.5 h, followed by two PBS washes to remove any non-adhered bacteria.

c. Displacement Test

In the DT, the processes were similar to the processes in the ET, with the differences being that the mixtures of *E. coli*-Susp and the same volume of PBS were first co-incubated with IPEC-J2 cells, followed by incubation with PSE and PBS mixtures.

The remaining steps were the same as for the previous three tests. A CO_2_-independent medium of 200 µL volume was added to ensure the growth of the adhered bacteria. The sigmoidal growth of *E. coli* was monitored at 650 nm as described for the AT.

### 2.6. Calculations and Statistical Analyses

The OD650 data from all of the miniaturised tests were analysed as described by Becker and Galletti [25], aiming to achieve a *t*OD = 0.05 value when the OD value of the cultured bacteria reached 0.05. Fitted equations established by our group were used to convert *t*OD = 0.05 values into colony-forming units (CFU) [8].

Extracts were tested at concentrations of 1% and 2% (*w*/*v*). All of the miniaturised tests included two independent assays per trial and were performed in triplicate, with a single assay as the experimental unit. The results are presented as means ± SD (log CFU). Significant differences between treatments were analysed using a linear model with two-way analysis (food and assays) of variance (ANOVA, R v.3.3). Significant differences between means were analysed using the Tukey–Kramer adjustment for multiple comparisons.

## 3. Results

The results of the AT showed different levels of adhesion to ETEC K88ac (Table 2). As the positive control, CGMP showed the highest ability to attach to the pathogen (*p* < 0.001) and EHB, EBR and ELM (2% and 1%) also showed similar levels of adhesion to ETEC K88 (*p* < 0.001). Extracts of shiitake at 2% and 1% exhibited higher adhesions than PBS (*p* < 0.01). No significant difference was detected for the aqueous extracts of jelly ear (EJE). Regarding the NF-*E. coli*, no food extracts showed a meaningful difference compared to the PBS group (Table 2).

In the CT, both EHB and EST at 2% (*p* < 0.01) and 1% (*p* < 0.05) significantly decreased the numbers of ETEC K88 attached to IPEC-J2 cells (Table 3). The positive group, CGMP, showed the lowest number of attached ETEC K88 (*p* < 0.001). Both EHB and EST at 2% had similar anti-adhesive results to the CGMP group. No significant difference was detected for EBR, ELM or EJE. Regarding the NF-*E. coli*, no food extracts showed a significant difference compared to the PBS group (Table 3).

In the ET (Table 4), none of the PSEs inhibited ETEC K88 from attaching to the IPEC-J2 cells. Regarding the NF-*E. coli*, no PSE showed a significant difference compared to the PBS group (Table 4).

In the DT (Table 5), none of the food extracts detached the attached ETEC K88ac from the IPEC-J2 cells. The positive control, CGMP, was able to remove more attached bacteria in the DT (*p* < 0.01). Regarding the NF-*E. coli* (Table 5), no food extract showed a significant difference compared to the PBS group, except for EJE at 2% and 1% (*p* < 0.05) with more detected NF-*E. coli*. There was no significant difference between the tested PSEs.

## 4. Discussion

ETEC K88ac pathogenic bacteria and IPEC-J2 cells were used in this study to mimic adhesin–host interactions. Four different in vitro assays were used to explore the anti-adhesive functions of some traditional foods containing high amounts of polysaccharides. Regarding the tested cereals, EHB, EBR and ELM specifically attached to more ETEC K88ac than the PBS group in the AT (Table 2), demonstrating that compounds in the water extracts of these ingredients could specifically connect with the adhesins of ETEC K88ac. Previous results from our group have also found this ability in different common cereals like oat, rye and wheat [8]. However, in the CT (Table 3), only EHB was able to reduce the bacterial attachment and no activity was reported in either the ET or in the DT. These results would suggest that EHB has the ability to specifically attach to adhesins and ultimately prevent ETEC K88ac from adhering to intestinal receptors. Results in the ET (Table 4) excluded the possibility of EHB efficiently blocking receptors on the epithelial cells. In the DT (Table 5), EHB failed to significantly remove attached ETEC K88ac from IPEC-J2 cells, indicating that it failed to form a higher affinity to the adhered pathogen. In previous studies by our group, barley was also found to recognise adhesins of ETEC K88 in the AT, but it did not significantly prevent the attachment of ETEC K88 in the CT [8]. Many factors probably contributed to the higher anti-adhesive activity of EHB in the CT and its higher content of β-glucan is worth investigating further in future research, despite the variable content of this bioactive compound within barley varieties [26,27].

Regarding shiitake, positive results in the AT (Table 2) and CT (Table 3) suggested that EST can bond with ETEC K88 and interfere with bacteria–cell adhesion. To the best of our knowledge, this is the first study to assess the potential anti-adhesive activity of shiitake, although several other studies with animal models have verified its potential in fighting intestinal pathogens. Studies conducted in chickens by Guo et al. showed that extracts of shiitake reduced caecal *E. coli* [28]. Moreover, in vivo studies with piglet models also described how dried shiitake (5%) efficiently reduced the number of *E. coli* in the stomach and jejunum [29].

As mentioned above, no ingredient showed significant results in the ET (Table 4) or in the DT (Table 5), illustrating that none of the tested ingredients was able to block receptors on IPEC-J2 cells or detach the attached ETEC K88ac from IPEC-J2. The positive results for EHB and EST in the CT suggest that some of the components of these ingredients could particularly bind with the ETEC K88 fimbriae and finally reduce the bacterial attachments to IPEC-J2 cells. Complex soluble carbohydrates, especially β-glucan, could be the bioactive compounds which are capable of providing analogue receptors to the fimbriae. This possibility is not a surprise, as previous studies have shown that ETEC K88 variants could recognise *N-acetylhexosamine* β-linked to a Gal residue and β1-linked galactosyl residues in intestinal glycoconjugate receptors [30,31]. It is also worth noting that the differences in the structures or types of glycosidic bonds and branches of β-glucans in the tested cereals and mushrooms may involve anti-adhesive processes [9,16,17]. Furthermore, regarding the β-glucans from shiitake and jelly ear, although they both consist of (1→3)-β-D-glucopyranoside units and β-(1→6)-d-glucosyl residues, their varied structures probably caused different anti-adhesive effects between EST and EJE [16,17]. Further studies are needed to verify this hypothesis, although more dissolved β-glucans in the PSEs were caused by the sonication process in this study. In addition, some other mechanisms are probably also involved in the anti-adhesive process. Recent studies have illustrated that dietary fibre could probably affect the attachment of ETEC to intestinal cells by impairing LT secretions as demonstrated by glucose [32,33].

In terms of the NF-*E. coli*, most of the PSEs in the assays showed similar results to the PBS groups, except for EJE in the DT (Table 5). After incubation, counts of the NF-*E. coli* in the EJE (2% and 1%) groups were even higher than those in the PBS group, but there were no differences with the other PSE groups. Further research is needed to explore the underlying mechanisms, including their anchor roles, although we hypothesise that some components of these soluble extracts had particularly promoted the growth of these bacteria.

## 5. Conclusions

In conclusion, the results from these experiments demonstrated that EHB, EBR, ELM and EST could offer receptors to ETEC K88ac adhesins. Moreover, EHB and EST would be able to provide receptor analogues to the K88ac fimbriae, explaining the reduction in the colonisation of IPEC-J2 by ETEC K88ac. These findings could illustrate, at least partially, some of the health benefits traditionally attributed to these ingredients. The results of this study could help to explore new functional properties of these ingredients in the prevention of diarrhoea caused by ETEC in humans and animals. The identification of different bioactive compounds, especially β-glucans, deserves to be investigated in future studies.

## Figures and Tables

**Table 1:** Analysed chemical composition of screened ingredients.

Ingredient	DM	Ash	CP	CF	EE	NDF	ADF	ADL	NFE
**Cereals**									
Highland barley	87.65	2.48	11.11	2.07	1.46	31.22	4.00	0.50	82.89
Little millet	87.67	0.81	11.10	0.25	1.93	3.10	1.28	0.10	85.91
Black rice	87.89	1.55	8.19	1.39	2.39	7.62	1.68	1.09	86.48
**Mushrooms**									
Shiitake	88.51	5.48	21.87	11.56	1.56	36.48	17.28	0.56	59.53
Jelly ear	86.41	4.59	14.38	15.54	0.94	ND	21.50	1.47	64.54

Chemical composition (g/100 g dry matter, except DM). DM: dry matter; CP: crude protein; CF: crude fibre; EE: ether extract; NDF: neutral detergent fibre; ADF: acid detergent fibre; ADL: acid detergent lignin; NFE: nitrogen-free extract; ND: Not Detected. NFE (%) = 100% − (Ash + CP + CF + EE).

**Table 2:** Number of bacteria (log CFU per well) that adhered to wells in the AT.

Test Extracts	Concentration	Incubated Bacteria	
		ETEC K88ac	NF-*E. coli*
PBS		6.67 ± 0.31 ^e^	7.57 ± 0.09
CGMP	1%	7.88 ± 0.03 ^a^	7.79 ± 0.09
EHB	2%	7.46 ± 0.02 ^bc^	7.69 ± 0.09
	1%	7.48 ± 0.03 ^abc^	7.40 ± 0.28
EBR	2%	7.38 ± 0.15 ^bcd^	7.59 ± 0.35
	1%	7.41 ± 0.08 ^bcd^	7.51 ± 0.42
ELM	2%	7.54 ± 0.07 ^ab^	7.64 ± 0.35
	1%	7.44 ± 0.15 ^bcd^	7.58 ± 0.33
EJE	2%	7.07 ± 0.12 ^cde^	7.60 ± 0.09
	1%	7.04 ± 0.01 ^de^	7.53 ± 0.12
EST	2%	7.33 ± 0.10 ^bcd^	7.82 ± 0.21
	1%	7.26 ± 0.14 ^bcd^	7.48 ± 0.16
SEM		0.103	0.199
*p*-Value		<0.001	0.332

PBS: phosphate-buffered saline; EHB: aqueous extracts of highland barley; EBR: aqueous extracts of black rice; ELM: aqueous extracts of little millet; EJE: aqueous extracts of jelly ear; EST: aqueous extracts of shiitake; CGMP: casein glycomacropeptide. Different superscript letters mean a significant difference (*p* < 0.05) among treatments.

**Table 3:** Number of bacteria (log CFU per well) that attached to IPEC-J2 in the CT.

Test Extracts	Concentration	Incubated Bacteria	
		ETEC K88ac	NF-*E. coli*
PBS		6.96 ± 0.09 ^a^	6.56 ± 0.02
CGMP	1%	6.12 ± 0.06 ^e^	6.37 ± 0.09
EHB	2%	6.42 ± 0.03 ^de^	6.59 ± 0.24
	1%	6.55 ± 0.05 ^cd^	6.48 ± 0.34
EBR	2%	6.68 ± 0.08 ^abcd^	6.52 ± 0.26
	1%	6.81 ± 0.10 ^abc^	6.92 ± 0.26
ELM	2%	6.83 ± 0.17 ^abc^	6.54 ± 0.19
	1%	6.95 ± 0.04 ^ab^	7.01 ± 0.42
EJE	2%	6.80 ± 0.15 ^abcd^	6.66 ± 0.32
	1%	6.77 ± 0.001 ^abcd^	6.66 ± 0.30
EST	2%	6.46 ± 0.11 ^cde^	6.22 ± 0.35
	1%	6.57 ± 0.14 ^bcd^	6.48 ± 0.30
SEM		0.097	0.252
*p*-Value		<0.001	0.206

PBS: phosphate-buffered saline; EHB: aqueous extracts of highland barley; EBR: aqueous extracts of black rice; ELM: aqueous extracts of little millet; EJE: aqueous extracts of jelly ear; EST: aqueous extracts of shiitake; CGMP: casein glycomacropeptide. Different superscript letters mean a significant difference (*p* < 0.05) among treatments.

**Table 4:** Number of bacteria (log CFU per well) that attached to IPEC-J2 in the ET.

Test Extracts	Concentration	Incubated Bacteria	
		ETEC K88ac	NF-*E. coli*
PBS		7.05 ± 0.02	6.37 ± 0.19
CGMP	1%	6.93 ± 0.10	6.26 ± 0.26
EHB	2%	7.07 ± 0.03	6.25 ± 0.26
	1%	7.05 ± 0.03	6.33 ± 0.14
EBR	2%	7.00 ± 0.03	6.31 ± 0.09
	1%	7.07 ± 0.14	6.32 ± 0.07
ELM	2%	7.06 ± 0.09	6.32 ± 0.11
	1%	7.07 ± 0.06	6.50 ± 0.10
EJE	2%	6.90 ± 0.11	6.55 ± 0.16
	1%	6.86 ± 0.17	6.45 ± 0.05
EST	2%	7.19 ± 0.04	6.37 ± 0.17
	1%	7.04 ± 0.23	6.55 ± 0.04
SEM		0.099	0.158
*p*-Value		0.188	0.607

PBS: phosphate-buffered saline; EHB: aqueous extracts of highland barley; EBR: aqueous extracts of black rice; ELM: aqueous extracts of little millet; EJE: aqueous extracts of jelly ear; EST: aqueous extracts of shiitake; CGMP: casein glycomacropeptide.

**Table 5:** Number of bacteria (log CFU per well) remaining attached to IPEC-J2 cells in the DT.

Test Extracts	Concentration	Incubated Bacteria	
		ETEC K88ac	NF-*E. coli*
PBS		7.17 ± 0.01 ^a^	5.79 ± 0.10 ^b^
CGMP	1%	6.58 ± 0.04 ^b^	6.18 ± 0.06 ^ab^
EHB	2%	7.33 ± 0.07 ^a^	6.27 ± 0.13 ^ab^
	1%	7.16 ± 0.02 ^a^	6.08 ± 0.29 ^ab^
EBR	2%	7.23 ± 0.09 ^a^	6.29 ± 0.30 ^ab^
	1%	7.09 ± 0.05 ^a^	6.28 ± 0.17 ^ab^
ELM	2%	7.16 ± 0.06 ^a^	6.35 ± 0.09 ^ab^
	1%	7.17 ± 0.06 ^a^	6.26 ± 0.29 ^ab^
EJE	2%	7.26 ± 0.32 ^a^	6.65 ± 0.31 ^a^
	1%	7.25 ± 0.12 ^a^	6.64 ± 0.05 ^a^
EST	2%	7.06 ± 0.13 ^a^	6.35 ± 0.21 ^ab^
	1%	7.11 ± 0.05 ^a^	6.45 ± 0.02 ^ab^
SEM		0.104	0.170
*p*-Value		0.002	0.017

PBS: phosphate-buffered saline; EHB: aqueous extracts of highland barley; EBR: aqueous extracts of black rice; ELM: aqueous extracts of little millet; EJE: aqueous extracts of jelly ear; EST: aqueous extracts of shiitake; CGMP: casein glycomacropeptide. Different superscript letters mean a significant difference (*p* < 0.05) among treatments.

## Data Availability

The original contributions presented in the study are included in the article, further inquiries can be directed to the corresponding author.
